# Identifying Unintended Stent Deformations in Aorto-Ostial Left Main Percutaneous Coronary Interventions

**DOI:** 10.1016/j.jscai.2025.103726

**Published:** 2025-06-10

**Authors:** Tinen L. Iles, Jens Flensted Lassen

**Affiliations:** a Department of Surgery, University of Minnesota, Minneapolis, Minnesota; b Department of Cardiology, Odense University Hospital & University of Southern Denmark, Odense, Denmark

**Keywords:** angiography, aorto-ostial lesions, bifurcation lesions, intravascular imaging, stent enhancement

Percutaneous coronary interventions (PCI) of aorto-ostial left main (LM) bifurcation lesions continue to increase worldwide and account for a significant amount of coronary bifurcation artery stenoses requiring stenting. The procedures are technically challenging, and despite the development of new generations of stent platforms with a more favorable risk profile, these interventions are still associated with higher risks of major cardiac events compared with nonbifurcation PCI.^1^ The increased risk of periprocedural myocardial infarctions and late adverse events is multifactorial, but suboptimal stent implantation and stent deformation caused by various stenting techniques that are not identified and corrected at the index procedure are the main causes of in-stent restenosis and stent thrombosis.^2^, ^3^, ^4^

PCI strategies for aorto-ostial LM lesions have developed over the years and have led to a recommended technique with uniform procedural steps.^2^, ^3^, ^4^ The complexity of the stenting techniques and the technical issues during implantation (eg, stent malapposition, unintended stent deformation [USD], longitudinal stent deformation [LSD], crushed stents, and undersized stents and floating stent struts over the side branch ostium) remain issues that correlate with higher rates of adverse late outcomes compared with nonbifurcation PCI procedures.^4^

In the OCTOBER trial (European Trial on Optical Coherence Tomography Optimized Bifurcation Event Reduction), USD was identified by the core lab in 18.5% of all cases with aorto-ostial LM bifurcation PCI, and guide catheter collision with the proximal stent edge and accidental abluminal rewiring were the most prevalent causes. The most striking finding was that USDs were not detected by the operator in half of the cases during the procedure, despite the trial being conducted in high-volume centers by dedicated operators with a special interest in optical coherence tomography (OCT). The 2-year rate of major adverse cardiac events for patients with untreated USD was 23.3%, whereas patients with correctly identified, confirmed, and corrected USD had no events during follow-up.^5^ These results underline the need for identifying USD during the procedure, where it can still be corrected. USD is particularly high in aorto-ostial LM bifurcation lesions due to the frequent need for rewiring, incorrect performance of bifurcation stent techniques, use of guide-extension catheters, multiple manipulations, and deep intubation with guiding catheters.^5^

Although challenging, the prevention and immediate identification of USD rely on careful preprocedural planning and meticulous conventional angiography-guided PCI. The key techniques include a thorough analysis of pre-PCI images (computed tomography angiography, multiple angiographic views, and quantitative coronary angiography vessel estimation), a systematic application of the technical steps suggested for a given technique, an intraprocedural or post-PCI use of stent enhancement, and a low threshold for use of intravascular imaging.^6^

The final evaluation of the results, however, still relies on angiography enhancement techniques and intracoronary imaging (ICI).^6^ Enhancement techniques, however, require different orthogonal views to fully assess the stent structure. ICI techniques (intravascular ultrasound or OCT) are currently considered the gold standard to identify USD, but OCT imaging remains difficult to perform in aorto-ostial lesions; a proper interpretation of the findings can be challenging even for trained operators, as shown in the OCTOBER trial.^5^ ICI is a powerful tool in planning the procedure, evaluating the procedural steps during the procedure, and documenting the final results, but ICI is not able to compensate for (unseen) suboptimal stent implantation.^5^ Accordingly, a more optimized approach to identifying USD is preferable.

In this issue of *JSCAI*, Amabile et al^7^ presented a new angiography-based technology that allows for visualization of stents in 3D online during the procedure (3DStent, GE HealthCare). It is based on a C-arm motion-compensated computed tomography principle and may improve the analysis of the stent structure and dimensions compared with conventional 2D stent enhancement tools. They investigated the diagnostic performance of the system on identifying LSD and pointed out that the most prevalent cause of LSD is guide catheter collision (or extension catheter collision) with the proximal stent edge. They elegantly compared 41 coronary stents implanted in silicone tubes where they created LSD by crushing the proximal stent edge with the guide catheter tip in half (n = 21) of the stents. Three independent reviewers blinded to the samples compared the 2 stent groups by angiographically based methods, including 3DStent imaging mode. They found very high overall sensitivity and specificity of LSD diagnosed by 3DStent imaging, which corresponds to a positive predictive value of 100% and a negative predictive value of 99%, and remarkably low inter- and intra-observer variabilities. The authors conclude that the 3DStent imaging technology could be used to efficiently assess LSD and state correctly that the technology needs to be tested in vivo as well before its routine use in the clinical setting. These findings have the potential to improve the diagnostic performance of angiography in identifying LSD/USD and may improve the angiographic diagnostic tools for identifying stent deformation and malapposition by making it possible to correct stent implantation during the procedure and improve the final long-term outcomes.

The following can be recommended to optimize aorto-ostial LM stenting: (1) Technical optimization of the bifurcation stenting procedures with a standardized, easy-to-follow description of the different technical steps in each recommended technique; (2) Introduction of new online angiography-based stent-enhancing techniques and ongoing training in optimal techniques and use of angio-guided PCI to avoid adverse procedural outcomes; (3) Extended use of ICI in aorto-ostial bifurcation stenting, and training to identify adverse procedural outcomes to facilitate immediate correction should they occur; and (4) Documentation of the final stent result with ICI.

Stent deformations left uncorrected carry a high risk of late events if not corrected. Continuous refinement of bifurcation stenting, aiming for an optimal result, is a major clinical need and can further reduce the risk of late adverse outcomes in aorto-ostial LM stenting.
